# Procedurepalooza: A Reproducible Workshop to Teach Core Procedures to Preclinical Learners

**DOI:** 10.5070/M5.53107

**Published:** 2026-07-31

**Authors:** Matthew Ryan

**Affiliations:** * University of Florida, Department of Emergency Medicine, Gainesville, FL

## Abstract

**Audience:**

This innovation is designed for first- and second-year medical students seeking early exposure to core procedural skills relevant to emergency medicine.

**Introduction:**

Early procedural training opportunities are limited in preclinical medical education, leaving students underprepared and lacking confidence in essential clinical skills.^1^ Simulation-based procedural instruction has proven effective for residents and senior students, but preclinical learners often lack similar access.^2^ Procedurepalooza was developed to address this gap by providing a scalable, low-cost, hands-on workshop introducing foundational procedural techniques in a supportive, high-engagement environment. Prior studies demonstrate that early procedural exposure improves confidence and skill acquisition; however, structured opportunities for preclinical learners remain limited.^3^

**Educational Objectives:**

By the end of the Procedurepalooza instructional session, learners will be able to: 1) recognize appropriate clinical indications for six foundational procedural skills commonly performed in emergency and acute care medicine, including intravenous (IV) placement, suturing, splinting, point-of-care ultrasound, airway management, and electrocardiogram (ECG) interpretation, 2) demonstrate proper technique in each procedure under guided supervision, emphasizing safety and ergonomics, and 3) perform core procedural steps through repeated hands-on practice in a structured small-group environment.

**Educational Methods:**

Procedurepalooza is a half-day, multi-station workshop teaching six essential procedural skills: intravenous (IV) placement, suturing, splinting, point-of-care ultrasound, airway management, and electrocardiogram (ECG) interpretation. Learners rotate through small-group stations (6–10 students) in 20-minute intervals, guided by faculty or senior residents. The workshop is designed for adaptability across institutions, requiring only basic equipment and reusable materials. The session was conducted as a live, hands-on workshop within the preclinical curriculum using a standardized small-group rotation model.^2^

**Research Methods:**

A pre- and post-event survey assessed self-reported procedural confidence across the six skills using a 5-point Likert scale. Participation was voluntary and anonymous. Quantitative analysis compared mean confidence ratings pre- and post-event.

**Results:**

Procedurepalooza has been conducted annually since 2012, consistently attracting strong student participation and faculty engagement. In one representative implementation, 148 students participated, and 102 completed both pre- and post-event surveys (70% response rate). Statistically significant improvements were observed across all procedural domains (*P* < 0.001). The largest gains occurred in intravenous (IV) placement (+1.80) and suturing (+1.68). Qualitative feedback emphasized the event’s accessibility, supportive teaching environment, and value in preparing students for clinical rotations.

**Discussion:**

Procedurepalooza significantly improved learner confidence in procedural skills through an engaging, reproducible, and low-cost format. While this event demonstrated improved procedural confidence, prior work shows that structured skills training enhances long-term procedural performance and that simulation-based education is associated with improved patient-related outcomes. Although we did not assess downstream proficiency, the combination of increased confidence and learner engagement suggests that early procedural exposure may support future clinical skill development.^4^,^5^ Its design promotes scalability and sustainability, making it suitable for institutions seeking to enhance preclinical procedural training with limited resources. Because Procedurepalooza also introduces preclinical students to emergency medicine earlier in their training—an experience typically deferred until later clinical years—this may anecdotally increase student interest in pursuing the specialty.

**Topics:**

Procedural skills, simulation, airway management, ultrasound, suturing, medical education innovation, emergency medicine.

## USER GUIDE

**Table:** 

List of Resources:	
Abstract	143
User Guide	145
Small Groups Learning Materials	149
Appendix A: Materials Checklist	151
Appendix B: Representative Procedural Checklists and Standardized Template	152
Appendix C: Procedurepalooza Student Survey	154
Appendix D: Sample Faculty Recruitment Email	156
Appendix E: ECGs	157

**Learner Audience:**

This innovation is designed for first- and second-year medical students seeking early exposure to core procedural skills relevant to emergency medicine.

**Time Required for Implementation:**

Faculty preparation requires approximately 2–3 hours and includes organizing materials, confirming space, and assigning instructors. Event setup on the day of implementation takes approximately 1–2 hours, depending on space and equipment availability, followed by a 3-hour instructional session. An additional hour should be allotted for post-event cleanup.

**Recommended Number of Learners Per Instructor:**

Each station is facilitated by one instructor for every 6–10 learners, with at least one instructor per station (6).

**Topics:**

Procedural skills, simulation, airway management, ultrasound, suturing, medical education innovation, emergency medicine.

**Objectives:**

By the end of the Procedurepalooza instructional session, learners will be able to:

1. Recognize appropriate clinical indications for six foundational procedural skills commonly performed in emergency and acute care medicine, including intravenous (IV) placement, suturing, splinting, point-of-care ultrasound, airway management, and electrocardiogram (ECG) interpretation.
2. Demonstrate proper technique in each procedure under guided supervision, emphasizing safety and ergonomics.
3. Perform core procedural steps through repeated hands-on practice in a structured small-group environment.

### Linked objectives and methods

The design of Procedurepalooza intentionally links its instructional objectives to established educational frameworks, including mastery learning, experiential learning, and social learning theory. Each method was selected to maximize skill acquisition, confidence, and engagement within the constraints of a single half-day event.

The small-group rotation format supports active learning by allowing students to alternate between observation and deliberate practice, a process central to mastery learning. Faculty and senior residents provide immediate, formative feedback to each learner, encouraging self-correction and repetition until confidence and procedural fluency improve. This structure directly supports the objective of enhancing procedural competence and comfort through measurable gains in self-assessed confidence.

The “see one, do one, do one again” approach draws from experiential learning theory, emphasizing repetition, reflection, and adaptation.^4^,^6^ Learners progress from passive observation to guided practice and finally to autonomous performance within the same session. This staged exposure reinforces muscle memory and strengthens retention, aligning with the objective of achieving measurable improvement in procedural confidence within the workshop timeframe.

Small-group, collaborative learning environments—including peer and near-peer interaction—are associated with increased learner engagement and the development of supportive, low-stakes learning conditions for procedural skill development.^2^,^4^ Faculty and senior learners model procedural technique, communication, and professional behaviors—such as maintaining patient comfort and dignity, using clear step-by-step explanations, and emphasizing safety and proper technique—while peers normalize early struggles and create a low-stakes learning environment. These interactions support the development of teamwork and communication skills in a hands-on, approachable setting.

Finally, pre- and post-event surveys provide quantitative assessment of learner growth (Appendix C) . This aligns with the objective of measurable improvement and supports ongoing quality improvement. Qualitative comments from participants offer insight into perceived value, helping refine subsequent iterations of the event.

Through these linked methods, Procedurepalooza operationalizes its goals in a way that is evidence-based, learner-centered, and easily reproducible across institutions with varying levels of resources.

### Recommended pre-reading for facilitator

No required pre-reading is necessary; however, facilitators should be familiar with the procedural skills they are teaching. To support reproducibility, suggested resources include procedural checklists for intravenous (IV) placement, suturing, splinting, airway management, point-of-care ultrasound, and ECG interpretation. Required equipment and materials are outlined in Appendix A, while representative checklists and a standardized template are provided in Appendix B to guide implementation.

### Learner responsible content (LRC)

No required pre-reading is necessary for learners. Procedurepalooza is designed as a self-contained, experiential workshop that welcomes all levels of preclinical students regardless of prior procedural exposure.

Optional preparatory materials may include brief instructional videos or one-page overviews of each skill^7^ provided via the institution’s learning platform. These resources allow motivated learners to familiarize themselves with equipment names and basic procedural steps before attending the live session.

### Small group application exercise (sGAE)

See the following attached materials for this small group exercise

- Appendix A: Materials Checklist
- Appendix B: Representative Procedural Checklists and Standardized Template
- Appendix C: Procedurepalooza Student Survey
- Appendix D: Sample Faculty Recruitment Email
- Appendix E: ECG

### Results and Tips for Successful Implementation

Procedurepalooza has been implemented annually since 2015 and was evaluated using pre- and post-event surveys (Appendix C) to assess learner confidence in six procedural domains: IV placement, suturing, splinting, airway management, point-of-care ultrasound, and ECG interpretation. In a recent implementation, a total of 148 students participated with 102 completing both surveys (70% response rate).

Mean confidence levels increased significantly across all six domains (*P* < 0.001), with the largest improvements observed in IV placement (+1.80) and suturing (+1.68) on a 5-point Likert scale. Qualitative comments consistently highlighted the value of early procedural exposure, approachable faculty, and the low-pressure learning environment. Table 1 summarizes pre-and post-event confidence gains across all procedural domains.

Implementation is most successful when the event is well-organized, materials are pre-staged, and instructor-to-learner ratios remain small (ideally 1:6–10). Scheduling Procedurepalooza midway through the preclinical year maximizes learner engagement because students have sufficient foundational knowledge to contextualize skills but limited real-world experience.

Several refinements have been made over time based on participant and instructor feedback:

- **Rotation time** was increased from 15 to 20 minutes per station to allow for repetition and coaching.
- **Station order** was reorganized to alternate high- and low-intensity procedures, balancing learner energy and attention.
- **Faculty briefing** now includes a 10-minute pre-event huddle to ensure consistency in instructional tone and key teaching points. During this huddle, facilitators review station-specific checklists (Appendix B), confirm equipment and setup using the materials list (Appendix A), align on key teaching priorities (e.g., proper technique, safety, and communication), and review the rotation schedule and learner flow.
- **Supply organization** was improved through standardized storage bins labeled by station, facilitating rapid setup and cleanup.

Overall, Procedurepalooza consistently achieves high satisfaction and measurable educational benefit. The event fosters enthusiasm for procedural learning and early engagement with clinical skill development among preclinical medical students.

Each station is ideally facilitated with one instructor for every six to ten learners, allowing for individualized guidance, direct observation, and immediate feedback. Small group sizes promote repetition and skill refinement in a low-pressure environment.

Instructors may include senior medical students, residents, fellows, or faculty with relevant procedural expertise. A mix of instructor levels enhances the experience, as near-peer instructors foster approachability while faculty provide clinical context. For a cohort of 100–150 learners across six stations, approximately 10–12 instructors allow for smooth rotation and minimize bottlenecks. A sample faculty recruitment email is provided in Appendix D.

Procedurepalooza was designed to be simple, portable, and resource-conscious. Most materials are reusable or readily available in simulation centers. Faculty preparation typically requires 2–3 hours and includes confirming space, organizing supplies, and assigning instructors to procedural stations.

On the day of the event, approximately one hour is required for setup, including arranging stations, placing signage, and ensuring equipment functionality. Once established, the event can be repeated annually with minimal additional preparation, since materials can be stored and reused.

The learner experience spans approximately three hours, during which students rotate through six stations in 20-minute intervals, allowing time for demonstration, hands-on practice, and feedback.

### Pearls for Learners

1. **Practice Deliberately**
  - Repeat each skill multiple times during the session.
  - Use feedback from instructors to adjust technique with each attempt.
2. **Focus on Core Technique**
  - Pay attention to hand position, angles, and step sequence.
  - Correct technique early—speed will come with repetition.
3. **Recognize When to Use the Skill**
  - Understand the basic clinical scenarios for each procedure (e.g., IV access, airway support).
  - Think about when you might encounter these skills during clinical rotations.
4. **Communicate Clearly**
  - Practice explaining what you are doing out loud.
  - Use simple, step-by-step language as if teaching a patient or teammate.
5. **Learn From Peers**
  - Watch others perform the procedure and note what works well.
  - Share tips and ask questions within your group.
6. **Build Confidence Through Repetition**
  - Confidence improves with practice, not perfection.
  - Identify one skill to revisit after the session for continued improvement.

### Take-Home Message

Procedural skill is not innate—it is *built*. Through structure, repetition, and teamwork, learners move from curiosity to capability.

### Suggested Ongoing Practice

- Review institutional or online procedural videos before clinical rotations.
- Use departmental checklists for IV placement and suturing during clerkships.
- Seek opportunities for deliberate practice in simulation or supervised clinical settings.

## Figures and Tables

**Table 1:** Pre- and Post-Event Procedural Confidence by Skill Station (n = 102)

Skill Station	Pre-Mean	Post Mean	Mean Change (Δ)	95% CI for Mean Change	P-value
IV Placement	2.05	3.85	+1.80	1.40–2.19	<0.001
Suturing	2.10	3.78	+1.68	1.25–2.11	<0.001
Splinting	2.25	3.65	+1.40	1.01–1.79	<0.001
Ultrasound	2.40	3.70	+1.30	0.92–1.68	<0.001
Airway Management	2.10	3.55	+1.45	1.05–1.85	<0.001
ECG Interpretation	2.43	3.54	+1.11	0.60–1.62	<0.001

## SMALL GROUPS LEARNING MATERIALS

### Small Group Application Exercise (sGAE)

Learners rotate through six procedural stations, each emphasizing deliberate practice and feedback. At every station, the instructor first demonstrates the skill, then guides students through hands-on repetition using the “see one, do one, do one again” approach.^4^ Learners work in teams of 6–10, alternating between performer and observer roles to reinforce muscle memory and peer learning. Pre- and post-event confidence surveys bookend the exercise, allowing reflection on individual growth.

**Stations:**

1. **Intravenous (IV) Placement** – Venipuncture on task trainer arms; focus on vein localization, proper technique, and catheter advancement.
2. **Suturing** – Simple interrupted and running sutures on silicone pads; focus on ergonomics and knot security.
3. **Splinting** application of a malleable aluminum splint (e.g., SAM^®^ splint) with soft immobilization using ACE wraps to a peer model; focus on stabilization and patient comfort.
4. **Airway Management** – Bag-valve-mask ventilation and basic intubation technique on airway manikin.
5. **Point-of-Care Ultrasound (POCUS)** – Probe handling and image acquisition on volunteer; focus on identifying basic structures.
6. **ECG Interpretation** – Pattern recognition and rhythm identification using laminated ECG strips representing common rhythms (e.g., normal sinus rhythm, atrial fibrillation, ventricular tachycardia, and ST-elevation myocardial infarction patterns). Please refer to the Appendix E for representative ECG examples and concise teaching descriptions for each rhythm.

To conclude the Procedurepalooza, learners complete the post-event survey at the conclusion of the final rotation and join a 10-minute debrief highlighting lessons learned and practical takeaways.

**sGAE Answers:**

- **IV Placement:** Identifies suitable vein, uses tourniquet, inserts catheter bevel-up, observes flashback, advances and secures.
- **Suturing:** Maintains needle angle ≈ 90°, achieves even tension, secures square knots.
- **Splinting:** Immobilizes joint above/below injury, verifies circulation post-application.
- **Airway:** Achieves chest rise with BVM, demonstrates correct head-tilt–chin-lift, recognizes proper tube depth.
- **Ultrasound:** Identifies target anatomy (vessel, gallbladder, bladder, etc.), maintains image orientation.
- **ECG:** Correctly recognizes rhythms such as sinus tachycardia, AFib, VT, VF, and STEMI equivalents.

**Brief Wrap Up:**

After all rotations, facilitators conduct a 10-minute debrief covering:

- Key take-home procedural principles.
- The importance of repetition for retention.
- Encouragement to seek continued practice during clinical rotations.
- Learners complete the post-event confidence survey and discuss how early exposure reduces anxiety and builds enthusiasm for clinical medicine.

## Appendix A Materials Checklist

**Table:**

Station	Supplies / Equipment Needed	Quantity / Notes
**IV Placement**	24 G 0.5-inch IV catheters	For ~100 students
	Band-aids	Ample supply
	Reusable tourniquets	Sufficient for all IV stations
	4x4 gauze	Ample supply
	Tape	Ample supply
	Gloves (Small, Medium, Large, X-Large)	Multiple boxes
**Suturing**	Sutures (3-0 and 4-0 Prolene, blue for visibility)	~100 sutures total
	Suture task trainers	~20 trainers
	Suture tool sets	One per trainer
**Splinting**	Splints, ACE wraps, other splinting materials	Sufficient for multiple rotations
**Airway Management**	Nasopharyngeal and oropharyngeal airways	Ample supply for practice
	LMAs and iGels	Ample supply for practice
	Intubating heads	4 heads (1 per table)
	Bougies, ET tubes (various sizes)	Multiple of each size
	Stylets, syringes	Ample supply
	C-MAC scope	1 scope
**Ultrasound**	Portable ultrasound machines	2 machines
**ECG Interpretation**	ECG printouts or machines	~12 printouts or machines

## Appendix B Representative Procedural Checklists and Standardized Template

**Representative Procedural Checklists**

1. **Intravenous (IV) Placement**^2^,^6^,^7^
  - **Indications:** Vascular access for fluids, medications, blood draws
  - **Equipment:** IV catheter, tourniquet, alcohol prep, gauze, tape, gloves
  - **Key Steps:**
    ○ Apply tourniquet and identify suitable vein
    ○ Clean site using aseptic technique
    ○ Insert catheter bevel-up at appropriate angle
    ○ Observe for flashback
    ○ Advance catheter and withdraw needle
    ○ Release tourniquet and secure catheter with dressing
  - **Safety Considerations:** Maintain sterility; avoid arterial puncture
  - **Common Pitfalls:** Poor vein selection, advancing without flashback, inadequate stabilization
  - **Teaching Tips:** Emphasize angle of entry, vein anchoring, and smooth advancement
  - **Suturing (Simple Interrupted)**^2^,^6^,^7^
  - **Indications:** Closure of simple lacerations
  - **Equipment:** Suture kit, needle driver, forceps, scissors, suture material, gloves
  - **Key Steps:**
    ○ Hold needle driver correctly and load needle
    ○ Enter skin at ~90° angle
    ○ Pass needle symmetrically through both wound edges
    ○ Tie secure square knots with appropriate tension
    ○ Cut suture tails to appropriate length
  - **Safety Considerations:** Avoid needlestick injury, maintain clean technique
  - **Common Pitfalls:** Uneven bites, excessive tension, poor knot security
  - **Teaching Tips:** Focus on wrist motion, spacing, and consistent knot tying
  - **Airway Management (Bag-Valve-Mask Ventilation)**^2^,^6^,^7^
  - **Indications:** Respiratory distress or failure requiring assisted ventilation
  - **Equipment:** Bag-valve-mask (BVM), oxygen source, airway adjuncts (oral pharyngeal airways (OPA) and nasal pharyngeal airways (NPA))
  - **Key Steps:**
    ○ Position patient and open airway (head-tilt–chin-lift or jaw thrust)
    ○ Select appropriate mask size and create a tight seal
    ○ Deliver ventilations with visible chest rise
    ○ Use airway adjuncts if needed
  - **Safety Considerations:** Avoid excessive ventilation, ensure proper seal
  - **Common Pitfalls:** Poor mask seal, inadequate airway positioning
  - **Teaching Tips:** Emphasize two-handed technique and teamwork when possible

**Standardized Procedural Checklist Template**

This template may be adapted for additional procedures (e.g., splinting, ultrasound, ECG interpretation):

- **Procedure Name:**
- **Indications:**
- **Equipment:**
- **Key Steps:**
- **Safety Considerations:**
- **Common Pitfalls:**
- **Teaching Tips:**

## Appendix C Procedurepalooza Student Survey

**Instructions:**

Please complete this survey to help us assess your experience. For each procedure listed, select your level of comfort *before* and *after* the event.

1. **Level of Comfort Assessment**
  **Table:**
  Procedure	Prior to Participating					After Participating				
  	Very Uncomfortable	Uncomfortable	Neutral	Comfortable	Very Comfortable	Very Uncomfortable	Uncomfortable	Neutral	Comfortable	Very Comfortable
  **IV Insertion**	□	□	□	□	□	□	□	□	□	□
  **ECG Interpretation**	□	□	□	□	□	□	□	□	□	□
  **Ultrasound**	□	□	□	□	□	□	□	□	□	□
  **Splint Application**	□	□	□	□	□	□	□	□	□	□
  **Suturing**	□	□	□	□	□	□	□	□	□	□
  **Airway Management**	□	□	□	□	□	□	□	□	□	□
2. **Most Valuable Station -** Which *one* station did you find the **most valuable**?
  **Table:**
  □ IV Insertion	□ ECG Interpretation	□ Ultrasound
  □ Splint Application	□ Suturing	□ Airway Management
3. **Prior Practice or Experience -** Please indicate which procedures you had **previous experience with** (check all that apply):
  **Table:**
  □ IV Insertion	□ ECG Interpretation	□ Ultrasound
  □ Splint Application	□ Suturing	□ Airway Management
4. **Career Interest -** Did participating in this event **increase your interest** in Emergency Medicine?
  **Table:**
  □ Yes	□ No	□ Unsure
5. **Event Feedback**
  What is one aspect of this event that could be improved?
  What was the best part about this event?
6. **Participant Demographics**
  **Table:**
  I am a:	MS1	MS2	MS3	MS4	Resident	Other___________________

## Appendix D Sample Faculty Recruitment Email

**Subject:** Help Teach Procedurepalooza! Faculty Volunteers Needed Dear Colleagues,

We are excited to announce this year’s *Procedurepalooza* — a hands-on procedural skills event for first- and second-year medical students, hosted by the Department of Emergency Medicine.

The event will take place on [date], from [time] in [location].

*Procedurepalooza* offers students early exposure to key clinical skills in a fun, supportive environment. Stations will include IV placement, suturing, splinting, airway management, ultrasound, and ECG interpretation.

We are seeking **faculty, fellows, and senior residents** to help teach at individual stations.

**No formal prep required** — just bring your clinical expertise and enthusiasm for teaching!

**Volunteer details:**

- Brief orientation provided before start
- Hands-on teaching at one station with small groups of students (6–10 learners at a time)
- Great opportunity to contribute to medical student education
- Standardized documentation of teaching participation will be provided for inclusion in promotion materials

We would love to have you join us!

Thank you for supporting this valuable educational event. Sincerely,

XXX

Department of Emergency Medicine

## Appendix E ECGs

The following ECG examples are intended for introductory rhythm recognition during Procedurepalooza small-group teaching sessions.

**Normal Sinus Rhythm**

Normal sinus rhythm demonstrates a regular rhythm with upright P waves preceding each QRS complex. The heart rate is typically between 60–100 beats per minute with a narrow QRS complex and consistent PR interval.

Image Attribution: Life in the Fast Lane Medical Blog. *Normal Sinus Rhythm ECG Library*. CC BY-NC-SA 4.0. Adapted and cropped for educational purposes. https://litfl.com/wp-content/uploads/2018/08/normal-sinus-rhythm-2.jpg

**Atrial Fibrillation**

Atrial fibrillation is characterized by an irregularly irregular rhythm without distinct P waves. Ventricular response is often rapid and variable, with narrow QRS complexes unless an underlying conduction abnormality exists.

Image Attribution: Life in the Fast Lane Medical Blog. *Atrial Fibrillation ECG Library*. CC BY-NC-SA 4.0. Adapted and cropped for educational purposes. https://litfl.com/wp-content/uploads/2018/08/ECG-Atrial-Fibrillation-4.jpg

**Anterior Myocardial Infarction**

Anterior myocardial infarction commonly demonstrates ST-segment elevation in the anterior precordial leads (V1–V4). Reciprocal changes may be present, and prompt recognition is critical because this pattern suggests acute coronary artery occlusion.

Image Attribution: Life in the Fast Lane Medical Blog. *Anterior Myocardial Infarction ECG Library*. CC BY-NC-SA 4.0. Adapted and cropped for educational purposes. https://litfl.com/wp-content/uploads/2018/08/ECG-Extensive-Anterior-STEMI-acute.jpg

**Atrial Flutter**

Atrial flutter classically demonstrates sawtooth flutter waves, most prominent in the inferior leads. Atrial rates are typically near 300 beats per minute with variable or fixed AV conduction producing ventricular rates such as 150 beats per minute.

Image Attribution: Life in the Fast Lane Medical Blog. *Atrial Flutter ECG Library*. CC BY-NC-SA 4.0. Adapted and cropped for educational purposes. https://litfl.com/wp-content/uploads/2018/08/ECG-Atrial-Flutter-with-Variable-Block.jpg

**Ventricular Tachycardia**

Ventricular tachycardia is a potentially life-threatening wide-complex tachycardia originating from the ventricles. The rhythm is usually regular with a heart rate greater than 100 beats per minute and wide QRS complexes.

Image Attribution: Life in the Fast Lane Medical Blog. *Ventricular Tachycardia ECG Library*. CC BY-NC-SA 4.0. Adapted and cropped for educational purposes. https://litfl.com/wp-content/uploads/2018/08/Monomorphic-ventricular-tachycardia-VT-3.jpg

**Ventricular Fibrillation**

Ventricular fibrillation demonstrates a chaotic, disorganized rhythm without identifiable P waves or QRS complexes. This rhythm produces no effective cardiac output and requires immediate defibrillation.

Image Attribution: Life in the Fast Lane Medical Blog. *Ventricular Fibrillation ECG Library*. CC BY-NC-SA 4.0. Adapted and cropped for educational purposes. https://litfl.com/wp-content/uploads/2018/08/ECG-Ventricular-fibrillation-VF-original-VF-VT.jpeg

**Supraventricular Tachycardia**

Supraventricular tachycardia is a regular narrow-complex tachycardia that usually originates above the ventricles. P waves are often absent or difficult to identify because of the rapid heart rate.

Image Attribution: Life in the Fast Lane Medical Blog. *Supraventricular Tachycardia ECG Library*. CC BY-NC-SA 4.0. Adapted and cropped for educational purposes. https://litfl.com/wp-content/uploads/2018/08/Slow-Fast-Typical-AVNRT.jpg

**Sinus Bradycardia**

Sinus bradycardia demonstrates a regular rhythm with upright P waves before each QRS complex and a heart rate less than 60 beats per minute. It may be normal in healthy individuals or associated with medications, vagal tone, or underlying illness.

Image Attribution: Life in the Fast Lane Medical Blog. *Sinus Bradycardia ECG Library*. CC BY-NC-SA 4.0. Adapted and cropped for educational purposes. https://litfl.com/wp-content/uploads/2018/08/ECG-Sin-us-bradycardia.jpg
